# A SYBR Green I based real time RT-PCR assay for specific detection and quantitation of *Peste des petits ruminants* virus

**DOI:** 10.1186/1746-6148-10-22

**Published:** 2014-01-14

**Authors:** Tsegalem Abera, Ardhanary Thangavelu, Navamani Daniel Joy Chandran, Angamuthu Raja

**Affiliations:** 1 College of Veterinary Medicine, Jigjiga University, Jijiga, Ethiopia; 2 Department of Veterinary Microbiology, Madras Veterinary College, Chennai 600007, India; 3 Department of Animal Biotechnology, Madras Veterinary College, Chennai 600007, India

**Keywords:** SYBR Green I, Real time RT-PCR, N gene, PPRV, Detection, Quantitation

## Abstract

**Background:**

*Peste des petits ruminants* (PPR) is an economically important disease of small ruminants such as sheep and goats. The disease is characterized by severe pyrexia, oculo-nasal discharge, pneumonia, necrosis and ulceration of the mucous membrane and inflammation of the gastro-intestinal tract leading to severe diarrhea. A SYBR Green I based real time RT-PCR targeting the N gene of PPRV has not been established for PPRV detection. Thus, the objective of present study was to develop highly sensitive N gene target SYBR Green I real time RT-PCR for specific detection and quantification of PPRV in clinical samples. A set of primers was designed to detect the nucleocapsid (N) gene of PPRV.

**Results:**

The assay exhibited high specificity as all the viruses which have clinical and structural similarities to PPRV including Canine distemper virus (CDV), Measles virus (MV), Bluetongue virus (BTV) and Newcastle disease virus (NDV) failed to show an amplification signal. The lower detection limit of the assay was 5.11 copies/μl (Ct value of 33.67 ± 0.5) and 0.001 TCID_50_/ml (Ct value of 34.7 ± 0.5) based on plasmid copy number and tissue culture infectivity titre. The assay was 3-log more sensitive than the conventional RT-PCR. The coefficient of variation (CV) values for intra- and inter-assay variability were low, ranging from 0.32% - 2.31%, and 0.71% - 5.32%, respectively. To evaluate the performance of the newly developed assay, a total of 36 clinical samples suspected of PPR were screened for the presence of PPRV in parallel with conventional RT-PCR. The real time RT-PCR assay detected PPRV in 30 (83.3%) of clinical samples compared to 16 (44.4%) by conventional RT-PCR.

**Conclusions:**

The two-step SYBR Green I based real time RT-PCR assay reported here is highly sensitive, specific, reproducible and rapid for detection and quantification of PPRV nucleic acids.

## Background

*Peste des petits ruminants* (PPR) is an economically important disease of small ruminants such as sheep and goats. The disease is characterized by severe pyrexia, oculo-nasal discharge, pneumonia, necrosis and ulceration of the mucous membrane and inflammation of the gastro-intestinal tract leading to severe diarrhea [1].

The causative agent of this disease, *peste des petits ruminants* virus (PPRV), is a member of the *Morbillivirus* genus in the *Paramyxoviridae* family. It has a negative sense single-stranded RNA genome encoding eight proteins [2]. The N gene of morbilliviruses is the most expressed gene due to a transcriptional gradient from the 3′ to the 5′ end of the genome and it is also well-conserved gene [3]. Thus, this gene is probably the best target for the development of a highly sensitive detection method.

Several conventional reverse transcription polymerase chain reactions (RT-PCRs) are available for detection of PPRV genomic material [4-6]. However, these conventional RT-PCR assays are labor intensive, as they require gel analysis for the detection of PCR products with a consequent high risk of contamination [7].

Real time RT-PCR has gained wider acceptance over conventional RT-PCR because it is more rapid, sensitive and reproducible. Few real time RT-PCR assays have been described for detection and quantification of PPRV in clinical samples using TaqMan chemistry [7-10]. In comparison with TaqMan-PCR, SYBR Green I based real-time RT-PCR assay has the advantages of being more cost-effective, easy to design, more precise and produced a more linear decay plot [11].

A single report described SYBR Green I based real-time RT-PCR assay targeting the M gene for detection of PPRV in clinical samples [12]. However, a SYBR Green I based real time RT-PCR targeting the N gene of PPRV has not been established for PPRV detection. In the present study, we developed highly sensitive N gene target SYBR Green I real time RT-PCR for specific detection and quantification of PPRV in clinical samples.

## Methods

### Viruses, cells and clinical samples

The different viruses used in this study are listed in Table 1. Vero cell adapted PPRV Coimbatore (CBE) field isolate, maintained in the Department of Veterinary Microbiology, Madras Veterinary College, Chennai 600 007, was used throughout this study to standardize the real-time RT-PCR assay. Viruses which have structural and/or clinical similarities to PPRV were included to test the specificity of the developed assay. Vero cells were used for virus propagation and titration. A total of 36 field samples, from PPR suspected outbreaks in different areas of the Indian state of Tamil Nadu, submitted to the Department of Veterinary Microbiology, Madras Veterinary College for diagnosis were analyzed. Samples received for analyses from different vet clinics were ocular, nasal, oral and rectal swabs, spleen, kidney, lung, lymph nodes and dung.

**Table 1:** Viruses used in the study

**Virus**	**Strain**	**Source**
Peste des petits ruminants virus (PPRV)	AR 87 vaccine strain	Dept. of Vet. Microbiology, Madras Veterinary College
	Sunguri vaccine strain	Indian Immunologicals Ltd, Bangalore
	Coimbatore vaccine strain	Dept. of Animal Biotechnology, Madras Veterinary College
	Coimbatore field isolate	Dept. of Vet. Microbiology, Madras Veterinary College
	Recent field isolates	Dept. of Vet. Microbiology, Madras Veterinary College
Canine distemper virus (CDV)	Vaccine strain	Nobivac Puppy DP, Intervet
Measles virus (MV)	Vaccine strain	Serum institute of India LTD, Pune
Bluetongue virus (BTV)	Vaccine strain	Dept. of Vet. Microbiology, Madras Veterinary College
Newcastle disease virus (NDV)	D58	Dept. of Vet. Microbiology, Madras Veterinary College

### Viral RNA extraction and cDNA synthesis

Viral RNA was extracted from tissue culture supernatant using QIAamp^®^ Viral RNA Mini Kit (Qiagen^®^, Germany), following manufacturer’s instructions. Trizol reagent (Invitrogen, CA, USA) was also used to isolate RNA from clinical samples. Synthesis of cDNA was carried out in 20 μl reaction using RevertAid™ H minus first Strand cDNA Synthesis kit (Fermentas, USA) following manufacturer’s instructions. Briefly, 11 μl of the purified RNA were added to a mixture containing 4 μl RT buffer (5×), 1 μl of random hexamer, 2 μl 10 mM dNTP, 1 μl RNase inhibitor and l μl of Moloney murine leukemia virus reverse transcriptase. The reaction was carried out at 25°C for 5 min, 42°C for 60 min and 70°C for 5 min and stored at -40°C until use.

### Primer design and synthesis

A set of primer was designed according to the sequences of nucleocapsid protein (N) gene (GenBank accession no. GQ452013.1) of PPRV Sungri-96 strain by using Fast PCR software. The designed primers (Forward N_2_F: 5′-GACGGCAT CAGGTTCAGGAG-3′ (57–76) and Reverse N_2_R: 5′-GCCAAT CTGACAAGCCTGTC G-3′ (156–177) were validated by OligoAnalyzer1.2 and synthesized commercially (Sigma Aldrich, Bangalore, India). The amplified cDNA fragment using primer pair N_2_F/N_2_R was expected to be 121 base pairs (bp) in length.

### Real time RT-PCR

The RT-PCR assay was performed on a Realplex^4^ real time PCR machine (Eppendorf, Germany) using SYBR premix Ex Taq, (TaKaRa Bio Inc., Japan). The assay was carried out in a total volume of 10 μl reaction mixture containing 5 μl of 2x SYBR Premix Ex Taq master mix, 1.5 μl of cDNA, 1 μl (10 μM) of each primer and 2.5 μl nuclease free water. The optimized cycling conditions were as follows: initial denaturation at 94°C for 5 min, followed by 40 cycles of denaturation at 94°C for 30 sec, primer annealing at 52°C for 30 sec, and extension at 72°C for 30 sec. The fluorescence was measured at the end of each cycle. A melt curve analysis was performed following amplification to verify the specificity of the amplified products. Melting curve analysis consisted of 70°C for 15 sec, and followed by temperature increase to 95°C for 15 sec at the rate of 1.25°C per sec with continuous reading of fluorescence.

### Conventional RT-PCR

For comparative purpose, conventional RT-PCR was performed on the cDNA preparations of the PPRV with a primer set of NP3 (5′-TCTCGGAAATC GCCTCACAGACTG-3′) and NP4 (5′-CCTCCTCCTGGTCCTCCAG AATCT-3′) as described by Couacy-Hymann *et al*., [5], which yielded 351 bp of PCR product. Each reaction tube of 20 μl contained 10 μl Taq DNA Pol 2.0x Master Mix Red (Biomol, Denmark), 1 μl (10 M) of each primer, 3 μl of cDNA and 5 μl of nuclease free water (Genei™, Bangalore, India). The mixture was subjected to an initial denaturation at 95°C for 5 min followed by 35 cycles of denaturation at 94°C for 30 s, annealing at 55°C for 30 s and extension at 72°C for 7 min. Amplification of target sequences was performed in a 2720 thermal cycler (Applied Biosystems). Amplification products (8–10 μl) were resolved on 1.5% agarose gel and stained with ethidium bromide for visualization.

### Generation of quantification standards

A fragment of 488 bp of N gene of PPRV containing the real time RT-PCR primers binding sites were amplified using the primer pair N_3_F (forward primer): 5′-CAAAGC GCCGA CGGCA TCA GGTT-3′ (48–70) and N_3_R (reverse primer): 5-′GCCAGAAGGATCCAGACTTGTGC-3′ (514–536) (In house designed). The RT-PCR product was cloned into RBC T&A cloning vector (RBC Biosciences) according to the manufacturer’s instructions. Plasmid DNA was recovered from the transformed *Escherichia coli* BL-21 cells using AxyPrep Plasmid Miniprep Kit (Axygen Biosciences). The OD value of the plasmid DNA standard concentrations was measured at 260 nm/280 nm on Thermo Scientific NanoDrop™ 1000 Spectrophotometer (NanoDrop Technologies, LLC, Wilmington, DE, USA). Plasmid copy number was calculated using the formula described by Adams [13].

### Specificity and sensitivity of the real time RT-PCR

The specificity of the developed assay was assessed against viral nucleic acid extracted from a range of animal viruses of clinical and structural relevance to PPRV. To check the sensitivity of the assay, PPRV CBE strain was titrated on Vero cells in a 96-well micro titre plate using standard cell culture procedure and the virus titre was calculated using Reed and Muench [14] formula. PPRV having a titre of 10^5^ TCID_50_/ml was diluted 10-fold serially from 10 ^-1 to 10 ^-11, and total RNA was extracted from each dilution, subsequently cDNA synthesis was performed. The synthesized cDNA was analyzed by newly developed two-step real-time PCR assay and simultaneously with conventional PCR to allow better comparison of the analytical sensitivity. Additionally, the sensitivity of the assay was determined by running 10-fold serial dilutions (5.11 × 10^7^ to 5.11 × 10^0^ copies/μl) of the plasmid standard in duplicates.

### Reproducibility

To evaluate reproducibility of the assay, the DNA standard ranging from 5.11 × 10^7^ copies/μl to 5.11 × 10^0^ copies/μl was tested repeatedly. Three separate dilution series were assayed in a single run to evaluate intra-assay variations. Whereas, the inter-assay variations were measured by testing each dilution in three separate consecutive runs. The mean, standard deviation (SD) and coefficient of variation (CV) for both intra-assay and inter-assay variations were calculated separately for each standard DNA dilution based on their Ct values using Microsoft Excel software.

### Spiking

To check the performance of the newly developed assay, clinical samples (2 nasal swabs from goats) were spiked with known titre of PPR virus as described by Balamurugan *et al*. [8]. Infected cell culture fluid containing 10^5^ TCID_50_/ml viruses was diluted 1: 10 serially by using the two negative nasal swabs as diluent. The 10^3^ and 10^4^ TCID_50_/ml spiked titre viruses were used for total RNA extraction. Control virus samples (10^3^ and 10^4^ TCID_50_/ml titre virus suspensions) were also used for RNA extraction.

### Ethical committee approval

Since we did not perform any trial by keeping experimental animals, ethical committee approval is not required as per our Institutional ethical committee. It means such work is ethically approved one which does not require specific submission of proposal and approval. TANUVAS Institutional Animal Ethical Committee Registration number is: No.190/CPCSEA/dated 22.05.2000. Our research work was approved by Institutional Biosafety Committee in its II meeting of the year 2011 dated 21.10.2011.

## Results

### Generation of standard curve

The concentration of the plasmid DNA was 176.2 ng/μl, which equates 5.11 × 10^10^ copies/μl. A series of 10-fold dilutions starting from 5.11 × 10^7^ to 5.11 × 10° were prepared. The correlation between the plasmid dilutions and the threshold cycle (Ct) values in real time RT-PCR were analyzed by plotting a standard curve. A linear regression relationship was observed with a coefficient of determination (R^2^) of 0.995, a slope of -3.1926 and a reaction efficiency of 105%. The generated standard curve covered a linear range of seven orders of magnitude and showed linearity over the entire range of quantification.

### Specificity

**Figure 1:**
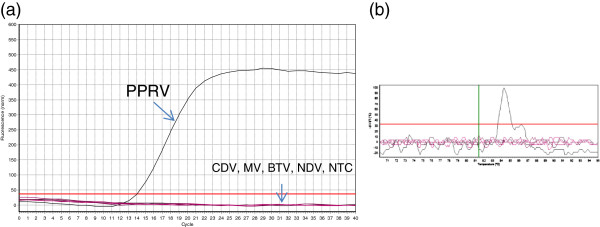
**Specificity of N gene based real time RT-PCR. (a)** amplification plot representing PPRV, CDV, MV, BTV, NDV and No template control (NTC). **(b)** Melting curve analysis.

Melting peaks analysis on the PCR products of plasmid DNA standard and 10-fold serially diluted tissue culture infected PPR virus showed that there were no primer-dimers and non-specific products. Only a single peak was visible in the melting peak chart. Specific amplification of the PPRV target sequence was identified by the generation of a melt peak at 84.31 ± 0.20. Specific amplification signal was found on available PPRV strains (Arasur, Sunguri, Coimbatore and recent field isolates). Additionally, the assay also exhibited specificity as an amplification signal was observed only with PPRV nucleic acid. None of the viruses (CDV, MV, BTV, and NDV) and no template control (NTC) showed an amplification signal (Figure 1).

### Detection limit

**Figure 2:**
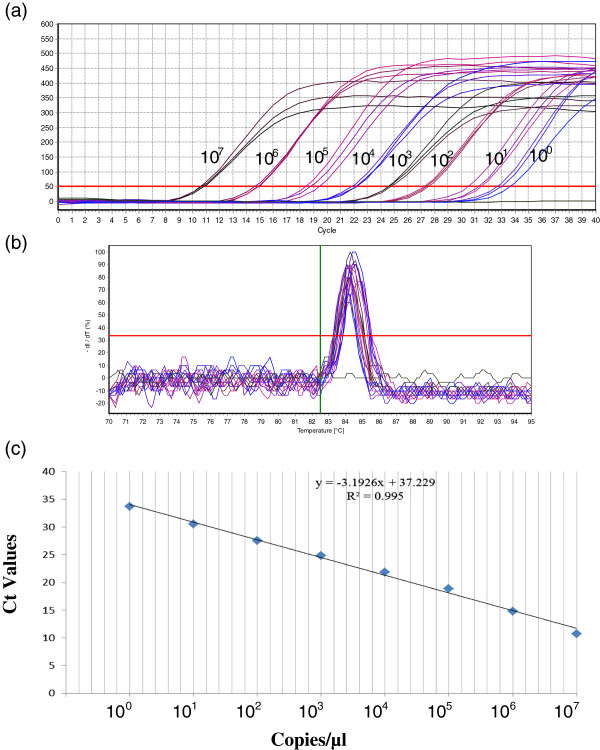
**The detection limit and standard curve of real time RT-PCR assay based on plasmid copy number. (a)** Amplification plot of 10-fold serial diluted plasmid DNA ranging from 5.11 × 10^7^ to 5.11 × 10° copies/μl. **(b)** Melting curve analysis. **(c)** Standard curve.

**Figure 3:**
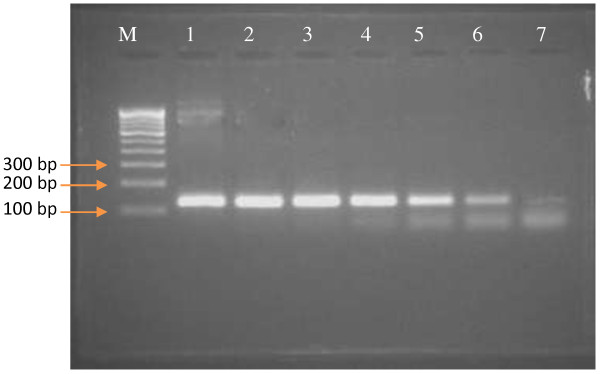
**Sensitivity based on plasmid copy number by using N**_**2**_**F/N**_**2**_**R primer pairs.** M-100 bp DNA ladder; Lane-1, 2.88 × 10^7^; Lane-2, 2.88 × 10^6^; Lane-3, 2.88 × 10^5^; Lane-4, 2.88 × 10^4^; Lane-5, 2.88 × 10^3^; Lane-6, 2.88 × 10^2^; Lane-7, 2.88 × 10^1^ copies/μl. product size, 121 bp.

**Figure 4:**
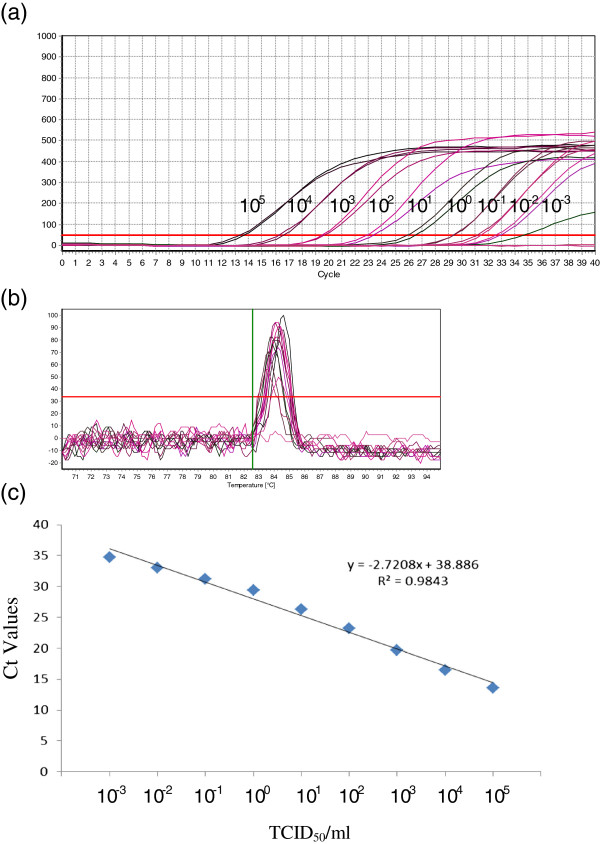
**Sensitivity of the real time RT-PCR based on serially diluted cell culture grown PPRV. (a)** Amplification plot of PPRV (from 10^5^ TCID_50_/ml to 0.001 TCID_50_/ml), **(b)** Melting curve analysis, **(c)** Standard curve.

The lower detection limit, based on plasmid copy number, achieved was 5.11 copies/μl with a corresponding Ct value of 33.67 ± 0.5 (Figure 2). At the same time, by using the same real time RT-PCR primer set in conventional RT-PCR the lower detection limit was found to be 5.11 × 10^1^ copies/μl (Figure 3). Further, the sensitivity of the assay was also evaluated by testing 10-fold serial dilutions of PPR virus having a titre of 10^5^ TCID_50_/ml. The assay could detect down to 0.001 TCID_50_/ml with corresponding Ct value of 34.7 (Figure 4).

### Comparison of SYBR Green I real time RT-PCR and conventional RT-PCR

**Figure 5:**
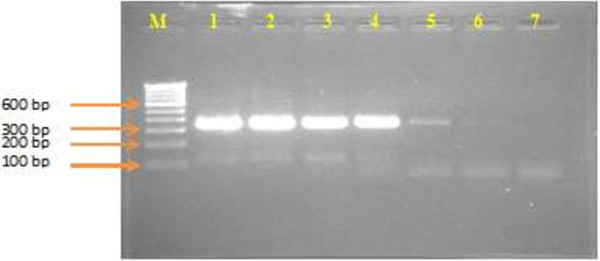
**Sensitivity of conventional RT-PCR based on TCID**_**50**_**/ml by using NP3/NP4 primer pairs.** Lane M-100 bp ladder; Lane-1, 10^5^ TCID 50/ml; Lane-2, 10^4^ TCID_50_/ml; Lane- 3, 10^3^ TCID_50_/ml; Lane-4, 10^2^ TCID_50_/ml; Lane-5, 10 TCID_50_/ml; Lane-6, 1 TCID_50_/ml; Lane-7, 0.1 TCID_50_/ml. Product size, 351 bp.

The detection limit of newly developed N gene based real time RT-PCR assay was compared with already established N gene based conventional RT-PCR assay (Couacy-Hymann *et al.*[5] by using a 10-fold serial dilutions of PPRV having a titre of 10^5^ TCID_50_/ml. The lower detection limit for SYBR Green I real time RT-PCR was found to be 0.001 TCID_50_/ml. However, the conventional RT-PCR assay (which amplified 351 bp) was able to detect 1 TCID_50_/ml (Figure 5). The SYBR Green I real time RT-PCR assay was found to be 1000 times more sensitive than conventional RT-PCR assay.

### Reproducibility

Reproducibility of the assay was assessed based on the Ct values obtained from testing DNA standard in triplicates. The intra-assay reproducibility of the assay was analyzed by using 10-fold dilutions of the plasmid DNA standard ranging from 5.11 × 10^7^ to 5.11 × 10^0^ copies/μl in triplicates per run. The calculated SD and CV values were, ranging from 0.10 to 0.58 and from 0.32% to 2.31%, respectively [Table 2(a)]. Whereas, the inter-assay variability was assessed by testing dilutions of the plasmid DNA standard in the range of 5.11 × 10^7^ to 5.11 × 10° copies/μl in three different experiments. The calculated SD and CV values were, ranging from 0.17 to 1.15 and from 0.71% to 5.32%, respectively [Table 2(b)].

**Table 2:** Reproducibility of PPRV N gene based real time RT-PCR assay

**a. Intra-assay variation**					
**DNA copies/μl**		**Ct values**		**Mean Ct**	**S.D**
5.11 × 10^7^	10.71	10.77	10.89	10.79	0.09
5.11 × 10^6^	14.52	14.81	14.75	14.69	0.15
5.11 × 10^5^	18.70	18.91	18.22	18.61	0.35
5.11 × 10^4^	21.77	21.75	21.58	21.70	0.10
5.11 × 10^3^	25.56	24.45	24.74	24.92	0.58
5.11 × 10^2^	27.07	27.27	26.89	27.08	0.19
5.11 × 10^1^	30.57	30.74	30.57	30.63	0.10
5.11 × 10°	32.52	33.26	32.59	32.79	0.41
**b. Inter-assay variation**					
**DNA copies/μl**		**Ct values**		**Mean Ct**	**S.D.**
	***Assay 1***	***Assay 2***	***Assay 3***		
5.11 × 10^7^	10.75	10.79	11.24	10.93	0.27
5.11 × 10^6^	14.84	14.70	16.17	15.24	0.81
5.11 × 10^5^	18.92	18.61	20.33	19.29	0.92
5.11 × 10^4^	22.07	21.7	23.14	22.30	0.75
5.11 × 10^3^	24.67	24.91	24.57	24.72	0.17
5.11 × 10^2^	27.42	27.08	27.61	27.37	0.27
5.11 × 10^1^	31.13	30.63	32.83	31.53	1.15
5.11 × 10°	33.12	32.79	33.74	33.22	0.48

### Spiking

Spiking assay with 10^4^ and 10^3^ TCID_50_/ml of cell culture infected PPRV was performed on two negative goat nasal swabs. The mean Ct values obtained for the spiked clinical samples and tissue culture virus were compared. Negative clinical samples spiked with known titre of PPRV and tissue culture virus with same titre were equally well detected by the assay.

### Evaluation of assay on clinical samples

To evaluate the performance of the SYBR Green-based real time RT-PCR assay for the detection of PPR virus in clinical samples, 36 samples from PPR suspected cases were analyzed. Comparison of the newly developed real time RT-PCR assay was made with gel-based conventional RT-PCR assay. Real time RT-PCR assay detected PPRV RNA in 30 (83.3%) samples. Only 16 (44.4%) samples were positive by N gene based conventional RT-PCR (Table 3).

**Table 3:** Comparative evaluation of clinical samples suspected of PPRV using conventional and real time RT-PCR assays

**Specimen**	**No.**	**Conventional RT-PCR positive**	**Real time RT-PCR positive**
Ocular swab	6	2	5
Nasal swab	9	7	9
Oral swab	3	1	2
Rectal swab	3	0	2
Lymph nodes	6	3	5
Lung	3	2	2
Kidney	1	0	1
Spleen	4	1	3
Dung	1	0	1
**Total**	**36**	**16**	**30**

## Discussions

A rapid, specific and sensitive diagnostic test is a must for the accurate diagnosis and control of this economically significant disease. In this study, a two-step SYBR Green I based real time RT-PCR assay targeting the N gene of PPRV was developed and evaluated in clinical samples.

The generated standard curve can be applicable for accurate quantification of PPRV. The assay maintained linearity over seven orders of magnitude. Compared with previously published real-time Taqman RT-PCR assay for the same gene target of PPRV [7,9,10] the present assay was more sensitive. This improvement in sensitivity may be due to the use of SYBR Green I chemistry. This is one of the main advantages of SYBR Green I chemistry over Taqman apart from its low cost and simpler approach in designing primers and optimization procedures [15]. The newly developed assay also performed better than the RT-PCR developed by Couacy-Hymann *et al*. [5] with respect to tissue culture infectivity titre.

The specificity of the assay was assessed in 4 different approaches: (1) by confirming the exact expected size of the amplicons in 2.5% agarose gel electrophoresis. The assay resulted in 121 bp product; (2) by melting curve analysis. Specific amplification of the PPRV target sequence was identified by generation of consistent melting peak at 84.31 ± 0.20. There was no primer-dimer or non-specific products; (3) by checking the assay for cross reactivity with a range of animal viruses of clinical and genetic similarities to PPRV. There was no cross reaction (no amplification) with any one of the viruses (CDV, MV, BTV, NDV); (4) by sequence analysis of the PCR product, which showed high identity with sequences of PPRV isolates available in GenBank. These results confirmed that the specificity of the designed primers in detecting PPRV genomic material.

The reported intra- and inter-assay variations with regard to Ct values were very small and they were comparable with previously reported Taqman based real time RT-PCR assays for PPRV [7,8]. This suggested that the new assay can generate reproducible results. The spiking assay showed that there was no measurable inhibition in the spiked negative nasal swabs when compared to pure tissue culture virus. This indicated that the PCR performance was the same in the spiked clinical samples and the tissue culture propagated virus.

The newly developed SYBR Green I based real time RT-PCR assay was validated on clinical samples. All the samples found positive by conventional RT-PCR were confirmed by real time RT-PCR. Additional positive samples were detected by the new assay. These results indicated that the assay developed in this study showed higher sensitivity than conventional RT-PCR in detecting PPRV nucleic acids directly from clinical samples.

## Conclusions

In conclusion, the two-step SYBR Green I based real time RT-PCR assay targeting the N gene of PPRV was highly sensitive, specific, reproducible and rapid for detection and quantification of PPRV nucleic acids. Since SYBR Green I is less expensive, this assay can be used in an economical manner. It can also be used as replacement for the existing Taqman RT-PCR for detection and quantitation of PPRV.

## Abbreviations

BTV: Bluetongue virus; CDV: Canine distemper virus; Ct: Threshold cycle; CV: Coefficient of variation; DNA: Deoxy ribonucleic acid; MV: Measles virus; NDV: Newcastle disease virus; PPRV: Peste des petits ruminants; RPV: Rinderpest virus; RT-PCR: Reverse transcription-polymerase chain reaction; SD: Standard deviation; TCID50: Tissue culture infective dose 50.

## Competing interests

Regarding competing interests we do have neither financial competing interests nor Non-financial competing interests.

## Authors’ contributions

TA: Developed the proposal, carried out the research and drafted the manuscript. AT: Participated in proposal development, advised the research and helped to draft the manuscript. NDJ: Participated in the design of the study and facilitated the work. AR: Designed primers and performed the statistical analysis. All authors read and approved the final manuscript.
